# Neochordameter: A New Technology in Mitral Valve Repair

**DOI:** 10.5812/cardiovascmed.12146

**Published:** 2013-10-28

**Authors:** Alireza Alizadeh-Ghavidel, Niloofar Samiei, Hoda Javadikasgari, Kamiar Bashirpour

**Affiliations:** 1 Heart Valve Disease Research Center, Rajaie Cardiovascular Medical and Research Center, Iran University of Medical Sciences, Tehran, IR Iran; 2 Mechanical Engineering Departments, Amirkabir University of Technology, Tehran, IR Iran

**Keywords:** Artificial Chordae, Mitral Valve Annuloplasty, Prolapse

## Abstract

**Background:**

Mitral valve repair has shown superior results compared to mitral valve replacement in patients with mitral valve prolapse. Using premeasured neochordae (the loop technique) has been proposed for both anterior and posterior leaflet repairs. However, there are two major problems that are usually experienced using this method. One is deciding the length of the neo-chordae, and the other is tying the knot at the intended length.

**Objectives:**

This study introduced a new technology in mitral valve repair that reduces the complexity of making neo-chordae loops, especially in minimally invasive surgeries.

**Patients and Methods:**

Neochordameter is a new device which utilizes preoperative transthoracic echocardiography to determine the exact length of required neochordae and enable surgeons to make neochordae loops before starting the cardiopulmonary bypass. In this study, we applied this technique in mitral valve repair of three patients.

**Results:**

Two of these patients were male and the other one was female. All of them had severe mitral regurgitation requiring anterior leaflet repair. Total eight neochordae loops were used in these patients. No change in the length of neochordae was required after saline test and all of these patients had none or trivial mitral regurgitation by intraoperative and follow up transesophageal echocardiography. No complication was seen in six-month follow up.

**Conclusions:**

The ability of this technology in developing premeasured neo-chordae loops with accurate sizes and not needing the post-implantation length adjustment which is efficient in reducing the complexity of both minimally invasive and conventional surgeries are the issues which is going to be regarded .

## 1. Background

Mitral valve prolapse is a common cardiac disease. Mitral valve repair (MVR) provided excellent midterm and long-term results and is considered the gold standard therapy for the patients with severe regurgitation (1). Using premeasured neo-chordae (the loop technique) has been proposed for both anterior and posterior leaflet repairs. However, there are two major problems that are usually experienced using this method. One is deciding the length of the neo-chordae, and the other is tying the knot at the intended length (2). In this study, we proposed a novel technique to address these issues.

## 2. Objectives

This study introduced a new technology in mitral valve repair that reduces the complexity of making neo-chordae loops, especially in minimally invasive surgeries.

## 3. Patients and Methods 

**Figure 1.**
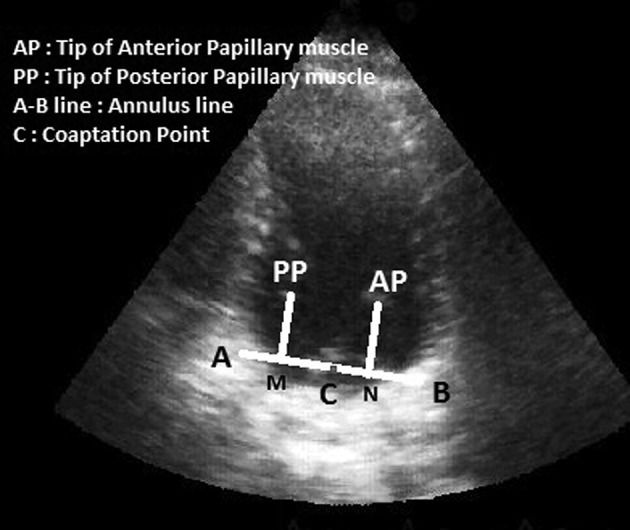
Two-Chamber View in Transthoracic Echocardiography

**Figure 2.**
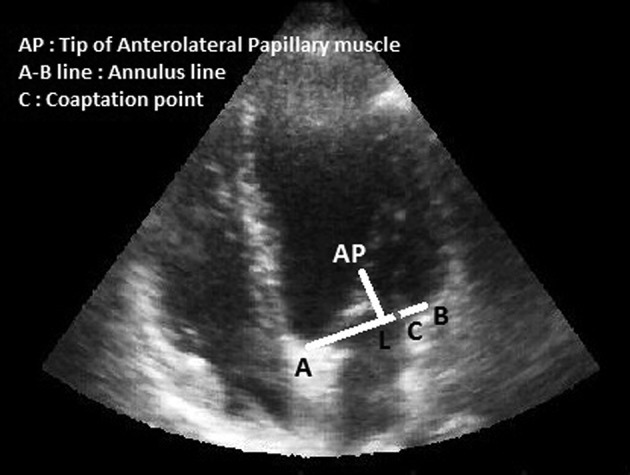
Four-Chamber View in Transthoracic Echocardiography

**Figure 3.**
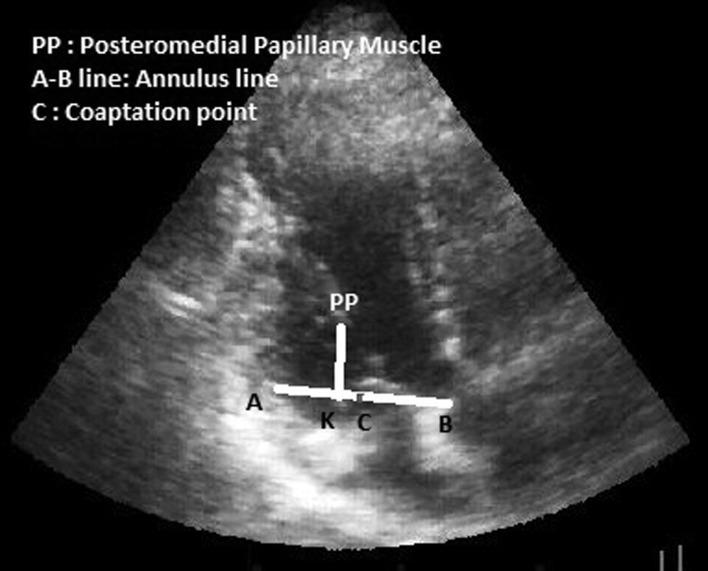
Apical Long Axis View in Transthoracic Echocardiography

**Figure 4.**
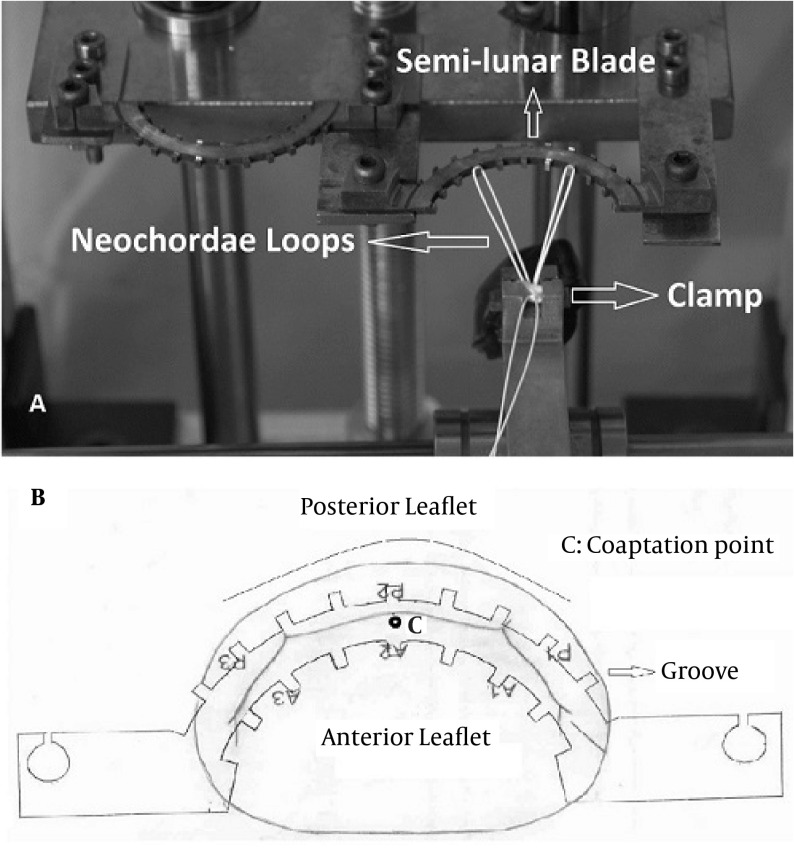
Neochordameter Elements

Neochordameter is a new device for making neo-chordae loops before starting the cardiopulmonary bypass. This device is setup using preoperative transthoracic echocardiography (TTE) views. We used TTE instead of transesophageal echocardiography (TEE) since left ventricular foreshortening is more serious in TEE rather than in TEE ( 3 , 4 ). Anterior/posterior, medial/lateral, and base to apex views provide the best images for determining the exact geometry of each patient’s left ventricular apparatus. Two- chamber view (medial/lateral view) provides the distance between the tip of each papillary muscle and the coaptation point (Figure 1). Four-chamber view provides the anterior distance between the tip of anterolateral papillary muscle and the coaptation point (Figure 2). Apical long axis provides the posterior distance between the tip of posteromedial papillary muscle and the coaptation point (Figure 3). Neochordameter is setup using these distances. A semi-lunar blade is designed in this device which models the free edge of anterior and posterior mitral valve leaflets (Figure 4 A). Several grooves are defined at each four millimeter distance on each free edge. They model the suture position on the free edge of patient’s mitral valve leaflets. A clamp (Figure 4 B) is designed for keeping the pledgete (considered the tip of the papillary muscle). The position of the clamp related to the center of semi-lunar blade can be changed (considered the coaptation point) based on the TTE measurements and enable the device to model the left ventricular geometry for each patient. This technique was applied for three patients who were candidate for MVR. The protocol of this study was approved by the Local Ethics Committee and a written informed consent was obtained from all patients. Neochordameter was set for each patient based on his/her individual preoperative TTE. Neochordae loops were made before starting cardiopulmonary bypass with regard to the position of prolapsed scallop in preoperative TTE. Saline test and intraoperative transesophageal echocardiography (TEE) were used to evaluate this technique. Ring annuloplasty was done for all patients. 

C-M and C-N lines demonstrate the distance between coaptation point and the tip of posteromedial and anterolateral papillary muscle, respectively.

C-L line demonstrates the anterior distance between coaptation point and the tip of anterolateral papillary muscle.

C-K line demonstrates the posterior distance between coaptation point and posteromedial papillary muscle.

## 4. Results

Three patients enrolled in the preliminary phase of this study. Two of these patients were male and the other one was female. All of them had severe mitral regurgitation requiring anterior leaflet repair. Total eight neochordae loops were used. Table 1 shows their pre and postoperative results. No change in the length of neochordae loops or any additional suture or modification were required after saline test. Intraoperative and follow-up TEE showed or trivial regurgitation or nothing. No complication was seen in three months follow-up. 

## 5. Discussion

Using neo-chordae for mitral valve repair has been favored by several surgeons from the first introduction (5-7). Since then, several methods have been proposed to facilitate estimating the length of required neo-chordae. These methods can be divided into two major categories. First one is determining the length of neochordae during operation and the second one is using preoperative echocardiography to find the length. However, all of these methods have shown some drawbacks including: measuring the length of neochordae in non-physiological state, the need for post-implantation length adjustment, and technical deficiencies for applying in minimally invasive and robotic surgeries. Matsui employed a new device consisting of two metallic tubes with a circular, hook shaped distal tip (8) Prêtre et al. (9) used an approach through the aortic valve for an anterior and posterior leaflet prolapse. They used saline test to examine adjustment of neo-chordal length. Tam et al. used a caliper during the surgery to measure the length of neochordae (10). Gillinov improved their methods and measured the length of neochordae with a caliper and made the loops around it (11). All of these mentioned methods are just applicable during surgery while the surgeon has not excellent view over the position of papillary muscles. Moreover, all of these methods require post-implantation length adjustment which further increases cardiopulmonary bypass time. Mandegar et al. used preoperative transesophageal echocardiography to estimate the distance between posterior papillary muscle and the free edge of the leaflet (12). Doi et al. used a combination of preoperative echocardiography and intraoperative measurements to estimate the length of neochordae (2). Recently, Matsui et al. used a new technique which needs only 12-French transparent plastic tube (13). Although these methods reduce the problem of intraoperative length measurement, both need post-implantation length adjustment. Our proposed technique has several advantages. All steps of this method including TTE and making the loops of neo-chorda are performed preoperatively in a physiologic state. Therefore, this would decrease the cardiopulmonary bypass time and increase the accuracy of length measurements. Furthermore, this ability would develop this technology for minimally invasive and robotic mitral valve repair.

**Table 1.** Patients’ Characteristics

Preoperative variables	Patient 1	Patient 2	Patient 3
**Age, y**	36	35	67
**Gender**	Male	Male	Female
**Body Mass Index **	26.84	26.64	30
**LVEF ^a^ (%)**	60	60	55
**End Systolic Volume, mL**	48	85	53
**End Diastolic Volume, mL**	80	204	150
**Left Atrial size, mm**	33	60	40
**IVRT ^a^**	65	70	60
**IVRT/ (QE - QE')**	13	14	12
**E /E' **	8.5	15	10.5
**Postoperative variables**			
**Ring size, mm**	32	32	32
**LVEF (%)**	55	50	50
**LVOT gradient ,mmHg**	4	4.2	5.7
**Coaptation length, mm**	6	6	6
**TMVG, mmHg**	4.6	4.2	2.26
**Number of neochordae**	3	3	2

^a^ Abbreviations: E, early ventricular filling velocity; E', peak diastolic velocity of the septal mitral annulus; LVEF, left ventricular ejection fraction; LVEF, isovolumic relaxation time; QE, the time interval from Q in the ECG to the onset of E; QE', the time interval from Q to the onset of E'; TMVG, trans mitral valve gradient
